# Hepatitis B virus and its sexually transmitted infection - an update

**DOI:** 10.15698/mic2016.09.527

**Published:** 2016-09-05

**Authors:** Takako Inoue, Yasuhito Tanaka

**Affiliations:** 1 Clinical Laboratory, Nagoya City University Hospital, Nagoya, Japan.; 2 Department of Virology & Liver unit, Nagoya City University Graduate School of Medical Sciences, Nagoya, Japan.

**Keywords:** Hepatitis B virus, Sexually transmitted infection, HIV/HBV coinfection, Genotype A, Hepatitis B vaccine

## Abstract

Epidemiology:* incidence and prevalence:* About 5% of the world’s population has chronic hepatitis B virus (HBV) infection, and nearly 25% of carriers develop chronic hepatitis, cirrhosis, and hepatocellular carcinoma (HCC). The prevalence of chronic HBV infection in human immunodeficiency virus (HIV)-infected individuals is 5%-15%; HIV/HBV coinfected individuals have a higher level of HBV replication, with higher rates of chronicity, reactivation, occult infection, and HCC than individuals with HBV only. The prevalence of HBV genotype A is significantly higher among men who have sex with men (MSM), compared with the rest of the population. Molecular mechanisms of infection, pathology, and symptomatology: HBV replication begins with entry into the hepatocyte. Sodium taurocholate cotransporting polypeptide was identified in 2012 as the entry receptor of HBV. Although chronic hepatitis B develops slowly, HIV/HBV coinfected individuals show more rapid progression to cirrhosis and HCC. Transmission and protection: The most common sources of HBV infection are body fluids. Hepatitis B (HB) vaccination is recommended for all children and adolescents, and all unvaccinated adults at risk for HBV infection (sexually active individuals such as MSM, individuals with occupational risk, and immunosuppressed individuals). Although HB vaccination can prevent clinical infections (hepatitis), it cannot prevent 100% of subclinical infections. Treatment and curability:* The *goal of treatment is reducing the risk of complications (cirrhosis and HCC). Pegylated interferon alfa and nucleos(t)ide analogues (NAs) are the current treatments for chronic HBV infection. NAs have improved the outcomes of patients with cirrhosis and HCC, and decreased the incidence of acute liver failure.

## INTRODUCTION

A sexually transmitted infection (STI) is defined as an infection that results from transmission of a pathogenic organism by sexual contact (i.e., any genital or anal contact with another person’s genitals, anus, or mouth) and that accounts for a noticeable amount of illness in the general population or in a defined subpopulation 1,2. Although there is no consensus on when the terms *STI* and *sexually transmitted disease *(*STD*) should be used, the American Sexual Health Association (ASHA) makes a distinction between the two terms 3. The concept of "a disease", as in STD, suggests a clear medical problem, usually some obvious signs or symptoms. However, most people infected with one or the other of several of the most common STIs do not manifest signs or symptoms, or have mild signs and symptoms that can be easily overlooked. A sexually transmitted virus or bacterium can infect its host, which may or may not result in "a disease". In this article, we use the term *STI*.

Organisms such as hepatitis B virus (HBV) that cause infections via sexual transmission can also cause infections via other routes, such as percutaneous transmission by contaminated needles and vertical transmission *in utero* or during delivery. As a typical STI, HBV infection is present in all types of populations. Sexual contact and vertical transmission from mother to infant are responsible for the large majority of HBV infections worldwide 4.

HBV infection as an STI is well documented. It is mainly common among men who have sex with men (MSM), because multiple partners are common in this population; and anal sex is usually more traumatic than vaginal intercourse, resulting in increased risk of exposure to blood 4,5. HBV infection is also extremely common among heterosexual individuals who have multiple sex partners or contact with sex workers 6.

Routine immunization with hepatitis B (HB) vaccine is strongly recommended for the prevention of HBV infection in MSM and other individuals at risk for STIs. HB vaccination of adults has been found to be effective at conferring immunity to individuals who are exposed to HBV via sexual transmission. However, the first priority is directly preventing the spread of HBV by the most reliable and appropriate method, which is use of a condom for safe sexual contact.

Chronic HBV infection is the cause of chronic hepatitis, cirrhosis, and hepatocellular carcinoma (HCC) 7. The goals of antiviral therapy for patients with chronic HBV are to slow the progression of chronic liver disease and decrease the development of complications, including cirrhosis and HCC. At present, pegylated interferon alfa (PEG-IFN-α), entecavir, and tenofovir disoproxil fumarate (tenofovir) are available for the treatment of HBV infection 8. Sodium taurocholate cotransporting polypeptide (NTCP) was recently identified as the receptor for HBV entry into hepatocytes 9. Because NTCP is essential for HBV infection, it may have potential as a new therapeutic target.

The purpose of this article is to provide up-to-date information on HBV and HBV infection as a major STI.

## ETIOLOGY AND MECHANISMS OF HBV INFECTION

### Etiology

#### Hepatitis B virus (HBV)

HBV is classified in the family *Hepadnaviridae*. It is a very small, partially double-stranded DNA virus. Humans are known to be the only natural host. HBV reaches the liver through the systemic circulation and can only replicate in hepatocytes 10. Since HBV is a hepatotropic virus, injury to the liver results from the immune-mediated destruction of infected hepatocytes 6.

The infectious HB virion has a diameter of 42-47 nm and is a double-shelled particle in serum. Its concentration can be as high as 108 virions per mL 6,10. The infectious HB virion consists of an outer lipoprotein coat (also called envelope) containing hepatitis B surface antigen (HBsAg). HBsAg surrounds an inner nucleocapsid composed of hepatitis B core antigen (HBcAg) that encapsidates the HBV genome and DNA polymerase 11,12.

#### Genome structure and proteins

**Figure 1:**
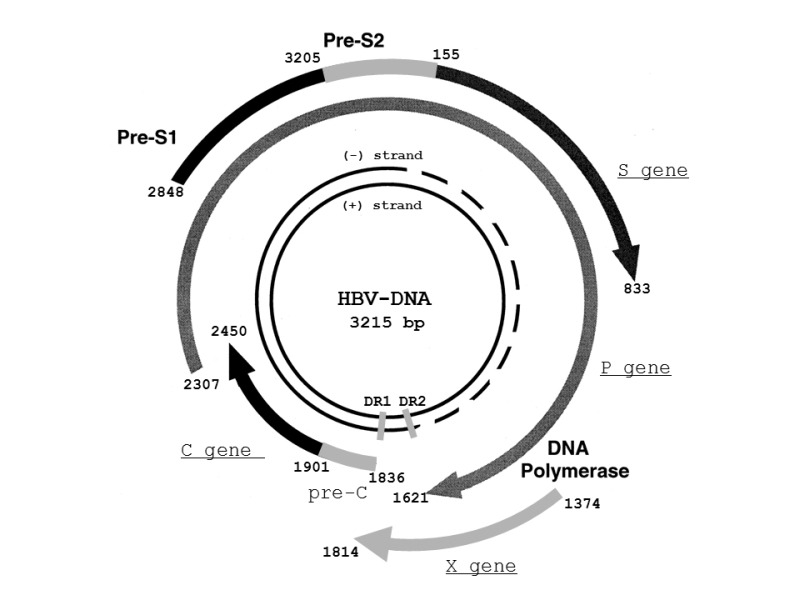
FIGURE 1: Structure and organization of the HBV genome 16. The four protein-coding regions are shown by semicircular arrows. They include the precore (pre-C) and core gene (C gene); the polymerase gene (P gene); the X gene; and the envelope genes pre-S1, pre-S2, and S (S gene). The positions of the direct repeats (DR1 and DR2) are indicated. Genome positions may change, depending on the HBV genotype 16. **Abbreviations:** HBV, hepatitis B virus; P gene, the polymerase gene; C gene, the core gene; S gene, the surface gene.

The HBV genome consists of a partially double-stranded, circular DNA molecule. Its total genome is 1700-2800 nucleotides long or 3020-3320 nucleotides long (for the short and full-length strand, respectively). Every nucleotide in the genome is active in 4 highly overlapping coding regions, or open reading frames (ORFs), as shown in Fig. 1 13,14,15,16. The polymerase gene (P gene) encodes the key enzyme for replication of the genome 13. The enzyme has DNA polymerase (DNA Pol), reverse transcriptase (RT) and RNase H activities, and also acts as the terminal protein (TP) 13,17. The core gene (C gene) has at least two in-frame start codons, and encodes HBcAg and HBeAg 13. HBcAg is the protein that encapsidates the viral DNA. It can also be expressed on the surface of hepatocytes, and evokes the cellular immune response 18. HBeAg is a marker of active viral replication 13. Secreted HBeAg is significantly more efficient than intracellular HBcAg at producing T-cell tolerance 19. The surface gene (S gene) encodes three different envelope glycoproteins, known as the pre-S1, pre-S2, and S proteins. The pre-S1 protein (large HBsAg) is the largest of the HBV surface proteins, and is produced starting at the first initiation codon of the ORF. The pre-S2 protein (middle HBsAg) is produced starting at the second initiation codon. The S protein (small HBsAg), which is commonly referred to as HBsAg or the Australia antigen, is produced starting at the third initiation codon. The X gene encodes the multifunctional X protein 13. It controls the level of HBV replication and acts as a cofactor in the development of HCC 20.

#### Natural history of HBV infection

HBV infection can cause acute hepatitis, acute liver failure, or chronic hepatitis, or can cause an asymptomatic infection. Chronic HBV infection can result in cirrhosis or HCC. The probability that a person with HBV infection will progress to chronic infection is strongly dependent on the person’s age at the time of HBV infection 21. More than 90% of HBV-infected infants and 25%-50% of children infected between the ages of 1 and 5 years will develop chronic hepatitis. More than 25% of HBV-infected infants and children older than 6 years will develop HBV-related cirrhosis and HCC 10. The rate of progression to cirrhosis and HCC is less than 1% per year for patients in the inactive chronic hepatitis stage, while the rate of progression to cirrhosis may be 2%-10% per year for patients in the immune active stage. By contrast, less than 10% of older children and adults with acute hepatitis progress to chronic infection. The progression from cirrhosis to HCC may occur in 2%-4% of adult patients per year 22.

In addition to age when first infected, the rates of progression of HBV infection are generally affected by gender, the level of HBV replication, HBV genotypes and variants, coinfecting viruses (hepatitis C virus [HCV], hepatitis delta virus [HDV], human immunodeficiency virus [HIV]), host lifestyle (drinking, smoking), exposure to carcinogenic substances, host genetic factors, and probably comorbidities (metabolic syndrome, diabetes and obesity) 22.

The natural history of chronic HBV infection can be separated into five stages, which are not necessarily sequential 23. These stages are summarized in Table 1 6.

**Table 1:** Stages of chronic HBV infection 6. Abbreviations: HBV, hepatitis B virus; HBeAg, hepatitis B e antigen; ALT, alanine aminotransferase; anti-HBe, antibody to hepatitis B e antigen; HCC, hepatocellular carcinoma; HIV, human immunodeficiency virus.

**Stage**	**HBeAg serological status**	**Pattern**	**Indications for treatment**
**1. “Immune tolerant”**	HBeAg positive	- Stage seen in many HBeAg-positive children and young adults, particularly among those infected at birth	Treatment not generally indicated, but monitoring required
		- High levels of HBV replication (HBV DNA levels >200 000 IU/mL))	
		- Persistently normal ALT	
		- Minimal histological disease	
**2. “Immune active” **(HBeAg-positive chronic hepatitis)	HBeAg positive; may develop anti-HBe	- Abnormal or intermittently abnormal ALT	Treatment may be indicated
		- High or fluctuating levels of HBV replication (HBV DNA levels >2000 IU/mL)	
		- Histological necroinflammatory activity present	
		- HBeAg to anti-HBe seroconversion possible, with normalization of ALT leading to “immune-control” stage	
**3. Inactive** **chronic hepatitis** **“Immune control”** (previously called inactive carrier)	HBeAg negative, anti-HBe positive	- Persistently normal ALT	Treatment not generally indicated, but monitoring required for reactivation and HCC
		- Low or undetectable HBV DNA (HBV DNA levels <2000 IU/mL)	
		- Reduced risk of cirrhosis and HCC	
		- May develop HBeAg-negative disease	
**4. “Immune escape” **(HBeAg-negative chronic hepatitis)	HBeAg negative, with or without anti-HBe positive	- HBeAg negative and anti-HBe positive	Treatment may be indicated
		- Abnormal ALT (persistent or intermittently abnormal)	
		- Moderate-to-high levels of HBV replication (HBV DNA levels >20 000 IU/mL)	
		- Older persons especially at risk for progressive disease (fibrosis/cirrhosis)	
**5. “Reactivation” or “acute-on-chronic hepatitis”**	HBeAg positive or negative	- Can occur spontaneously or be precipitated by immunosuppression from chemo- or immunosuppressive therapy, HIV infection, or transplantation; development of antiviral resistance; or withdrawal of antiviral therapy	Treatment indicated
		- Abnormal ALT	
		- Moderate to high levels of HBV replication	
		Seroreversion to HBeAg positivity can occur if HBeAg negative	
		- High risk of decompensation in presence of cirrhosis	

##### Stage 1: "Immune tolerant"

The initial stage represents the incubation period. When HBV is actively replicating, HBV DNA, HBeAg, and HBsAg are detected in the serum 24. The serum alanine aminotransferase (ALT) is only slightly or not elevated, and the infected person is not symptomatic. The immune response is limited to production of antibody to hepatitis B core antigen (anti-HBc) (immunoglobulin M [IgM] followed by immunoglobulin G [IgG]); however, these antibodies do not neutralize the infection 25. This first stage occurs more frequently and has a longer duration in babies infected during delivery or during the first years of life 23. There are only few or no findings of fibrosis. In this stage, though treatment is not generally indicated, monitoring is required.

##### Stage 2: "Immune active" (HBeAg-positive chronic hepatitis)

HBeAg can be detected in the serum. A somewhat lower level of HBV DNA is seen in some patients, who are clearing HBV, than in stage 1 24. Compared with the previous stage, the serum ALT level is higher, and there is moderate or severe liver necroinflammation and more rapid progression of fibrosis 23,26,27,28. For patients with chronic HBV infection, 10 years or more may pass before cirrhosis develops, immune clearance takes place, or HCC develops. The immune response reduces the level of HBV replication, and begins to clear HBeAg and HBsAg. The rate of development of antibody to hepatitis B e antigen (anti-HBe) and HBeAg clearance (HBeAg seroconversion) is 10%-20% per year. Chronic infection will develop in 80%-90% of infected infants 29, whereas less than 5% of infected adults will fail to resolve acute hepatitis 30. This stage ends with HBeAg seroconversion 23. In this stage, treatment may be indicated.

##### Stage 3: Inactive chronic hepatitis "immune control" (previously called inactive carrier)

The stage of inactive chronic hepatitis may follow the seroconversion to anti-HBe and clearance of HBeAg. The stage is characterized by very low or undetectable HBV DNA in the serum and serum aminotransferase levels in the reference range 23. Through immunological control of HBV infection, the majority of patients will have a favorable outcome with very low risk of cirrhosis or HCC 31,32. HBsAg is still present in the serum, but HBsAg clearance and development of antibody to hepatitis B surface antigen (anti-HBs) may occur spontaneously in 1%-3% of cases per year 33. In this stage, although treatment is not generally indicated, monitoring for reactivation and HCC is required.

##### Stage 4: "Immune escape" (HBeAg-negative chronic hepatitis)

The HBeAg-negative chronic hepatitis stage may follow clearance of HBeAg and development of anti-HBe during the inactive chronic infection stage (stage 3) or directly from the immune active/clearance stage (stage 2). It is important to distinguish inactive HBV carriers from individuals negative for HBeAg who have chronic hepatitis. The former patients will have a good outcome with a very low risk of complications, while the latter have a high risk of progressive liver disease, including decompensated cirrhosis and HCC 23. In this stage, treatment may be indicated.

##### Stage 5: "Reactivation" or "acute-on-chronic hepatitis"

In the final stage, HBV reactivation may occur spontaneously or may be triggered by cancer chemotherapy or other immunosuppressive therapies, and may result in serious acute-on-chronic hepatitis. Occult HBV infection is defined as persistence of HBV DNA in the liver of individuals in whom HBsAg is undetectable in the blood. Individuals who have cleared HBsAg and are negative for serum HBV DNA but anti-HBc positive may develop reactivation if they are being treated with potent immunosuppressive medications 6.

HBsAg loss before the onset of cirrhosis is associated with improved outcome, with a reduced risk of cirrhosis, decompensation, and HCC 23. If cirrhosis develops before natural or treatment-induced clearance of HBsAg, patients remain at risk of HCC 34. In this stage, treatment is indicated.

### HBV life cycle

NTCP was recently identified as a receptor for HBV entry, which enabled the establishment of a susceptible cell line that can efficiently support HBV infection. This discovery should lead to a deeper understanding of the requirements for effective HBV infection and clarification of the molecular mechanism of HBV entry.

The replication cycle of HBV begins with entry of the virus into hepatocytes, which is mediated by the binding of the pre-S1 region on the virion envelope to the hepatocellular NTCP 13. The virion is then uncoated and transported into the nucleus. The viral relaxed circular DNA (rcDNA) or linear DNA genome, with a protein attached to the 5’ end of the minus strand and a short RNA attached to the 5’ end of the plus strand 35, is converted into covalently closed circular DNA (cccDNA) through covalent ligation 14.

This cccDNA is responsible for viral persistence and is highly resistant to antiviral therapy. It serves as the template for the transcription of viral mRNAs. The pregenome mRNA serves for the synthesis of core protein (nucleocapsid subunit) and viral reverse transcriptase. The viral genome is replicated by reverse transcription of pregenomic RNA. During this process, both the protein and the RNA are removed 35. The reverse transcriptase binds to the 5’ end of its own mRNA template, and the complex is then packaged into nucleocapsids, where viral DNA synthesis occurs. These nucleocapsids can also move into the nucleus to increase the copy numbers of cccDNA. Since cccDNA does not undergo semiconservative replication, all cccDNA copies result from viral DNA made in the cytoplasm via the reverse transcription pathway 36.

**Figure 2:**
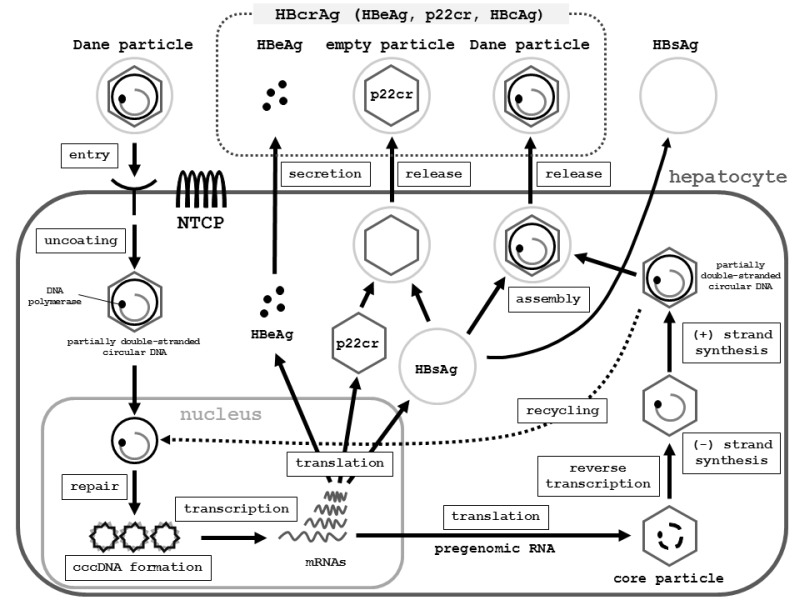
FIGURE 2: Schematic representation of the HBV lifecycle, from entry into hepatocytes to release from hepatocytes. **Entry: **HBV (Dane particle) obtains entry into hepatocytes by binding to the receptor NTCP 37,38,39 and possible additional hepatocyte-specific factors on the cell surface. The HBV membrane fuses with the membrane of the host hepatocyte, and the virion is endocytosed. **Uncoating: **The HBV membrane releases the viral DNA (partially double-stranded circular DNA) with the core particle into the cytoplasm 39. The viral membrane is lost (uncoating). The viral nucleocapsid containing the viral genomic DNA is transported into the nucleus in the relaxed circular form. **Repair and cccDNA formation: **In the nucleus, the viral DNA polymerase synthesizes fully double-stranded DNA, and fully double-stranded DNA is converted to a cccDNA by the viral DNA polymerase 38,39. The formation of cccDNA remains poorly understood. It is most likely formed via the DNA repair mechanism 38. **Transcription: **cccDNA is transcribed into the pregenomic and subgenomic mRNAs by host RNA polymerase 38,39. **Translation and reverse transcription: **Pregenomic RNA is the template for the translation of both DNA polymerase and the core proteins, and for reverse transcription. The DNA polymerase binds to the packaging signal of the pregenomic RNA, and both are then combined into the viral capsid, which is the core particle 38,39. The HBV genome matures in the core particle via reverse transcription of pregenomic mRNA to DNA 39. **DNA synthesis: **After synthesis of the (-) strand DNA and (+) strand DNA, the nucleocapsid, containing partially-double stranded circular DNA, is generated. **Assembly: **HBsAg and the nucleocapsid containing partially double-stranded circular DNA are assembled together to become a new complete virion 39. **Release: **The mature HBV virion (Dane particle) is released from the infected hepatocyte or is recycled back into the nucleus for amplification of cccDNA 38. **Other events: **The C gene directs the synthesis of two major gene products: HBcAg (p21c), which comprises the nucleocapsid; and HBeAg (p17e), which is a secreted antigen. Noninfectious particles (empty particles), which are composed of HBsAg, a 22-kDa precore protein (p22cr), and HBeAg, are also produced as a trap for the host immune system, in order to protect the infectious Dane particles. Serologic testing can assess HBeAg, p22cr, and HBcAg as hepatitis B core-related antigen (HBcrAg). **Abbreviations:** HBV, hepatitis B virus; NTCP, sodium taurocholate cotransporting polypeptide; cccDNA, covalently closed circular DNA; RC-DNA, relaxed circular DNA; HBsAg, hepatitis B virus surface antigen; HBcAg, HBV core antigen; HBV e antigen, HBeAg; p22cr, precore protein; HBcrAg, hepatitis B core-related antigen.

An increase in the level of viral envelope proteins inhibits synthesis of high levels of cccDNA, which can be toxic to hepatocytes. Once partially double-stranded DNA has been produced, nucleocapsids can undergo a maturation event that enables them to obtain an outer envelope via budding into the ER. The mature nucleocapsids may be recycled into the nucleus to mediate viral persistence, or secreted as Dane particles through exocytosis to infect other hepatocytes 35 (Fig. 2) 37,38,39.

## EPIDEMIOLOGY: INCIDENCE AND PREVALENCE

### Incidence: worldwide view and HIV/HBV coinfection

More than one third of the world’s population are estimated to be infected with HBV. About 5% of the world’s population are chronic carriers of HBV, and HBV infection causes more than one million deaths every year 40. The HBsAg carrier rate varies from 0.1% to 20% of different populations worldwide. In low-risk regions, the highest incidence of infection is seen in teenagers and young adults.

Based on the data from Western cohorts, HIV/HBV coinfection has a profound impact on almost every aspect of the natural history of HBV infection 6. The consequences include higher rates of chronicity after acute HBV infection, higher levels of HBV replication and rates of reactivation, less spontaneous clearance, higher rates of occult HBV infection (i.e., detectable HBV DNA positivity in the absence of HBsAg seropositivity), more rapid progression to cirrhosis and HCC, higher rates of liver-related mortality, and decreased treatment response compared with individuals without HIV coinfection 41,42. Recent longitudinal cohort studies have found that coinfection with HBV also can lead to increased rates of progression to acquired immune deficiency syndrome (AIDS)-related outcomes and all-cause mortality 43,44. An estimated 5% to 15% of the 34 million HIV-infected individuals worldwide are coinfected with HBV, as a chronic infection 45,46. The burden of coinfection is greatest in Southeast Asia and sub-Saharan Africa 6.

### Prevalence: international statistics

An estimated 240 million people are chronically infected with hepatitis B 47. The prevalence of chronic HBV infection varies geographically, ranging from 1% to 20%. Populations with high rates include Alaskan Eskimos, Asian-Pacific islanders, Australian aborigines, and populations of the Indian subcontinent, sub-Saharan Africa, and Central Asia. In some locations, such as Vietnam, the rate is as high as 30% 48. The prevalence of the HBV carrier state is related to differences in the mode of transmission, including iatrogenic transmission, and the age of primary infection.

In low-prevalence (< 2%) regions, the lifetime risk of HBV infection is less than 20%. Sexual transmission and percutaneous transmission during early adulthood are the main routes of spreading the infection. About 12% of HBV-infected individuals live in the low-prevalence regions, which include North America, northern and western Europe, Australia, and New Zealand 48. In these areas, most HBV infections occur in adolescents and young adults belonging to relatively well defined high-risk groups, which include injection drug users, MSM, healthcare workers, and patients who undergo regular blood transfusions or hemodialysis 48,49.

In intermediate-prevalence (3% - 5%) regions, sexual and percutaneous transmission and vertical transmission during delivery are the major routes of infection. These regions include eastern and southern Europe, Japan, the Mediterranean basin, the Middle East, Latin and South America, and Central Asia. One study reported that approximately 43% of HBV-infected individuals live in southern, central, and western Asia; eastern Europe; Russia; and Central and South America. The lifetime risk of HBV infection is 20% - 60% 48. The persistently high rates of chronic infection are mostly due to infections occurring in infants and children.

In high-prevalence (10% - 20%) regions, transmission occurs predominantly in infants and children. During early childhood, HBV is transmitted vertically from the mother to infant or occurs via close contact. In some regions, percutaneous exposure to contaminated needles or an unsafe injection is also a possible route of HBV infection. Since most infections in children are asymptomatic, there is little evidence of acute HBV-related disease, but the rates of chronic liver disease and HCC in adults are high. Approximately 45% of individuals infected with HBV live in high-prevalence regions. The lifetime risk of infection is higher than 60%, as demonstrated by the presence of anti-HBc in sera 48. The high-prevalence regions are mostly regions with developing economies and large populations. They include China, Southeast Asia, Indonesia, sub-Saharan Africa, the Pacific Islands, parts of the Middle East, and the Amazon Basin 48.

### HBV serotypes and genotypes

Based on some of the antigenic determinants of HBsAg, nine serological types - referred to as subtypes*adw2*, *adw4*,* adrq+*, *adrq*, *ayw1*, *ayw2*, *ayw3*, *ayw4* and *ayr* - have been identified 50. Ten genotypes of HBV (A-J) have been identified, and these correspond to specific geographic distributions 51. Genotype A is more frequently found in North America, northwestern Europe, India, and Africa. Genotypes B and C are endemic to Asia, and genotype D predominates in eastern Europe and the Mediterranean 52. Type E is found in western Africa; type F, in South America; and type G, in France, Germany, Central America, Mexico, and the United States. Type H is prevalent in Central America 48; type I, in Vietnam; and type J (possible recombination with type C), in Japan 53.

HIV-seropositive MSM populations predominantly coinfected with HBV genotype A have been reported in European countries and Japan 54,55,56. The prevalence of HBV genotype A is significantly higher in the MSM population than in the rest of the population 56. In addition, Araujo *et al.* speculated in their review that HBV subgenotypes A2 and C are likely to predominate in populations at high risk of infection via sexual transmission 57. Additionally, HBV genotype A develops into a persistent infection more often than genotype C 58,59.

Individuals infected with genotypes C and F have higher rates of HCC than individuals infected with genotypes B and D 6. Evidence increasingly suggests that genotype C and F affect disease severity and response to treatment 60,61,62. Results of studies in Asia suggest that patients infected by HBV genotype C show a more rapid progression to cirrhosis and HCC than patients infected by genotype B 63,64,65, and subgenotypes of HBV genotype C are probably responsible for the increased rate of HCC in patients who were positive for HBeAg 66. Studies in Europe and North America have found that higher proportions of patients with chronic hepatitis associated with genotype D infection progressed to cirrhosis and HCC than those with chronic hepatitis associated with genotype A infection 26,67,68,69.

## PATHOPHYSIOLOGY / SEROLOGY / SIGNS AND SYMPTOMS

### Pathophysiology

#### Pathological findings

Currently, most liver biopsies are performed to confirm the existence of chronic hepatitis and to determine its level of activity. This section mainly describes chronic hepatitis, which plays an important role in HBV infection.

##### 1) Acute hepatitis B

Because acute hepatitis B is always diagnosed by clinical symptoms and serologic markers related to HBV infection, liver biopsies are not often performed. In general, acute hepatitis shows more areas of spotty parenchymal inflammation and more severe damage than typical chronic hepatitis. The lesions mainly contain diffuse sinusoidal and portal mononuclear infiltrates (lymphocytes, plasma cells, Kupffer cells), swollen hepatocytes and/or necrotic hepatocytes (also called apoptotic or acidophilic hepatocytes, or Councilman bodies) 70,71. Cell plates and sinusoids may be indistinct in more severe cases as a result of hepatocyte swelling, filling of sinusoids by mononuclear inflammatory cells, and regenerating hepatocytes. Significant lobular necrosis leads to acute liver failure 70.

##### 2) Chronic hepatitis B and cirrhosis

In chronic HBV infection, there is a varying degree of predominantly lymphocytic portal inflammation with interface hepatitis and spotty lobular inflammation. The inflammation is minimal in the immune-tolerant or inactive chronic infection stages, but is prominent in the immune-active stage. Bridging necrosis is identified as inflammation "connecting" portal tracts to one another or to central veins 71. Confluent necrosis affects multiple contiguous hepatocytes. Inflammation is typically associated with scarring, which can vary from a mild portal extension to periportal fibrous strands, bridging fibrosis, and cirrhosis. Livers that develop central to portal bridging necrosis or confluent necrosis are likely to have a higher fibrosis stage. The Scheuer classification for grading and staging of chronic hepatitis is often used, as shown in Table 2 72.

**Table 2:** Scheuer classification for grading and staging of chronic hepatitis 72.

**Grade**	**Portal / periportal activity**	**Lobular activity**
0	None	None
1	Portal inflammation	Inflammation but no necrosis
2	Mild piecemeal necrosis	Focal necrosis or acidophil bodies
3	Moderate piecemeal necrosis	Severe focal cell damage
4	Severe piecemeal necrosis	Damage includes bridging necrosis
**Stage**	**Fibrosis**	
0	None	
1	Enlarged, fibrotic portal tracts	
2	Periportal or portal-portal septa, but intact architecture	
3	Fibrosis with architectural distortion, but no obvious cirrhosis	
4	Probable or definite cirrhosis	

The hepatocytes that express a high level of HBsAg may have a "ground-glass" cytoplasm, which can be highlighted by special immunohistochemical stains (Shikata’s orcein and Victoria blue). Ground-glass hepatocytes may also be seen in other conditions 73.

Cirrhosis is diagnosed when the loss of normal central-portal relationships is observed. The atypical enlargement of nuclei with an increase in the nuclear-cytoplasmic ratio, known as "large cell change", is very common in cirrhosis. This cytologic abnormality should only be used to support the evidence of regeneration and architectural abnormalities, which is used for diagnosing cirrhosis 70.

### Diagnosis

#### Serologic markers related to HBV infection

The serologic markers of HBV infection are as follows: HBsAg and the corresponding antibody anti-HBs, HBeAg and the corresponding antibody anti-HBe, immunoglobulin M antibody to hepatitis B core antigen (IgM anti-HBc), immunoglobulin G antibody to hepatitis B core antigen (IgG anti-HBc), and serum HBV DNA. The diagnosis of acute or chronic HBV infection requires serologic testing (Table 3) 2. The first detectable markers in acute HBV infection are HBsAg and IgM anti-HBc.

**Table 3:** Interpretation of results of serologic tests for HBV infection 2. Symbol for negative test result, “-“; symbol for positive test result, “+.” Abbreviations: HBV, hepatitis B virus; HBsAg, hepatitis B surface antigen; anti-HBc, antibody to hepatitis B core antigen, IgM anti-HBc, immunoglobulin M antibody to hepatitis B core antigen; HBIG, hepatitis B immune globulin.

**Serologic marker**				**Interpretation**
**HBsAg**	**Total anti-HBc**	**IgM anti-HBc**	**Anti-HBs**	
–	–	–	–	Never infected
+	–	–	–	Early acute infection, transient (up to 18 days) after vaccination
+	+	+	–	Acute infection
–	+	+	–	Acute resolving infection
–	+	–	+	Recovered from past infection and immune
+	+	–	–	Chronic infection
–	+	–	–	False positive (i.e., susceptible), previous infection, “low-level” chronic infection, passive transfer to infant born to HBsAg-positive mother
–	–	–	+	Immune if concentration is >10 mIU/mL, passive transfer after HBIG administration

Total anti-HBc is present over the entire lifetime of the infected individual. It is found in individuals with chronic HBV infection and in those who recover from HBV infection 10. The presence of anti-HBc alone might indicate acute, resolved, or chronic infection, or a false-positive result 2. HBsAg and HBeAg can be used as surrogate markers of HBV replication 74. HBsAg is eliminated from the sera of individuals who recover from HBV infection, and anti-HBs is detectable during recovery 10. Detection of HBsAg indicates early acute infection. To ensure that an HBsAg-positive test result is not false positive, samples with repeatedly reactive HBsAg results should be tested with a US Food and Drug Administration (FDA)-cleared neutralizing confirmatory test 2. HBeAg is a marker of high levels of viral replication. Detection of HBeAg indicates that the blood and body fluids of an infected person are highly infectious. Detection of anti-HBeAg indicates inactive chronic hepatitis. The persistence of HBeAg for longer than 10 weeks and/or HBsAg and serum HBV DNA for longer than 6 months, indicates transition to chronic HBV infection 74. Detection of anti-HBs indicates immunity against HBV. Anti-HBs can also be detected in individuals who were immunized by the HB vaccine. Most individuals who recover from HBV infection are expected to be positive for both anti-HBs and anti-HBc 10. Individuals positive for anti-HBc only are unlikely to be infectious, except under unusual circumstances, including direct percutaneous exposure to large quantities of blood (e.g., blood transfusion and organ transplantation) from individuals positive for anti-HBc only 2.

#### Acute hepatitis B

The incubation period (duration from exposure to HBV to onset of symptoms) of HBV-infected individuals with acute hepatitis ranges from 60 to 150 days, with an average of 90 days 75,76. The signs and symptoms of acute hepatitis B are described in detail in the “Signs and symptoms” section. As mentioned previously, the clinical manifestations of acute HBV infection are age dependent 10. Over 90% of infants with HBV infection are asymptomatic, while the typical manifestations of acute hepatitis are prominent in 5% to 15% of newly infected young children (aged 1-5 years) and in 33% to 50% of children older than 6 years of age 10,21. Serologic markers related to acute hepatitis B are described in the subsection*“*Serologic markers related to HBV infection*”.* As described in that subsection, the persistence of HBeAg indicates the transition to chronic HBV infection 74.

#### Chronic HBV infection

The natural history of HBV infection, including the transition to chronic infection, is described in the “Etiology” section. Chronic HBV infection is defined as either the presence of HBsAg in the serum for at least 6 months or the presence of HBsAg in a person who tests negative for IgM anti-HBc 10. Unlike individuals who recover from acute HBV infection, patients with chronic HBV infection do not produce anti-HBs, and serum HBsAg positivity typically persists for a long period of time 10. In patients with chronic HBV infection, the disappearance of HBeAg and detection of anti-HBe usually indicate a reduction in viral load 77. Each year, approximately 0.5% of adults with chronic HBV infection will clear HBsAg and produce anti-HBs 78,79,80. Although patients with chronic HBV infection die of causes unrelated to HBV, chronic HBV infection is responsible for most of the morbidity associated with HBV 10. Follow-up studies of individuals first infected with HBV when they were infants or young children, show that approximately 15% to 25% of patients with chronic infection die prematurely from cirrhosis or HCC 81,82.

### Signs and symptoms

#### Symptoms and physical findings

The manifestations of HBV infection during the acute phase vary from subclinical hepatitis to acute hepatitis and acute hepatic failure. During the chronic phase, disease progression varies from asymptomatic chronic infection to chronic hepatitis, cirrhosis, and HCC 83. The findings on physical examination vary from minimal to remarkable, according to disease severity. The signs, symptoms, and findings on physical examination are listed in Table 4.

**Table 4:** Manifestations of hepatitis B virus infection.

**Clinical staging**	**Signs, symptoms, and clinical finding**
Acute hepatitis	General fatigue, loss of appetite, nausea, vomiting, abdominal pain, low-grade fever, jaundice, hepatomegaly, splenomegaly, palmar erythema, spider nevi, Gianotti-Crosti syndrome (papular acrodermatitis), serum-sickness-like syndrome, necrotizing vasculitis (polyarteritis nodosa), membranous glomerulonephritis (MGN), cryoglobulinemia, aplastic anemia, transient maculopapular rash.
Chronic hepatitis	Similar to acute hepatitis, (hepatomegaly, splenomegaly, muscle wasting, palmar erythema, spider angioma, vasculitis).
Progressive liver disease, including hepatic decompensation	Ascites, jaundice, history of variceal bleeding, peripheral edema, gynecomastia, testicular atrophy, abdominal collateral veins (caput medusa), hepatic encephalopathy, somnolence, disturbances in sleep patterns, mental confusion, coma, variceal bleeding, coagulopathy, pleural effusion, hepatopulmonary, and portopulmonary syndrome.
Acute liver failure	Ascites, fever, jaundice, hepatomegaly, splenomegaly, hepatic encephalopathy, somnolence, disturbances in sleep pattern, mental confusion, coma, variceral bleeding, coagulopathy.

Acute hepatitis B is an illness that begins with general fatigue, loss of appetite, nausea, vomiting, body aches, low-grade fever, dark urine, and jaundice. The illness lasts for several weeks and then gradually improves in most affected individuals. A few individuals may develop more severe liver disease (acute hepatic failure) and may die. In addition, acute hepatitis B infection may be entirely asymptomatic and may go unrecognized 84.

Some acute hepatitis B patients (about 1%) may develop acute liver failure, which is characterized by evidence of decompensated liver disease and is fatal in up to 50% of cases 83. Patients with acute liver failure can present with the following signs and symptoms: hepatic encephalopathy, somnolence, disturbed sleep patterns, mental confusion, coma, ascites, variceal bleeding, and coagulopathy.

Individuals with chronic HBV infection may be asymptomatic or may manifest the signs and symptoms associated with chronic hepatic inflammation. Patients with chronic active hepatitis, especially during the replicative stage, can manifest symptoms similar to acute hepatitis (fatigue, anorexia, nausea, and mild upper quadrant pain or discomfort). Physical examination of patients with chronic HBV infection can reveal the typical characteristics of chronic liver disease, including hepatomegaly, splenomegaly, muscle wasting, palmar erythema, spider angioma, and vasculitis.

In cases with progressive liver disease, the following manifestations may be present: hepatic decompensation, hepatic encephalopathy, somnolence, disturbed sleep patterns, mental confusion, coma, ascites, variceal bleeding, coagulopathy, ascites, jaundice, peripheral edema, gynecomastia, testicular atrophy, and collateral abdominal veins (caput medusa). Pleural effusion and hepatopulmonary and portopulmonary syndrome may occur in patients with cirrhosis. Patients with cirrhosis may have the following findings: ascites, jaundice, history of variceal bleeding, peripheral edema, gynecomastia, testicular atrophy, and collateral abdominal veins.

#### Extrahepatic manifestations

Extrahepatic manifestations of HBV infection occur in 1% to 10% of patients, and include serum-sickness-like syndrome, acute necrotizing vasculitis (polyarteritis nodosa), membranous glomerulonephritis (MGN)^85^, and papular acrodermatitis of childhood (Gianotti-Crosti syndrome) 86,87. Serum-sickness-like syndrome occurs in the setting of acute hepatitis B, often preceding the onset of jaundice 88. The manifestations often subside shortly after the onset of jaundice, but can persist throughout the duration of acute hepatitis B 11. About 30% to 50% of people with acute necrotizing vasculitis (polyarteritis nodosa) are HBV carriers 89. HBV-associated nephropathy has been described in adults but is more common in children 90,90. MGN is the most common form. Other immune-mediated hematological disorders, such as essential mixed cryoglobulinemia and aplastic anemia, can also occur 11.

A variety of cutaneous lesions can appear during the early course of viral hepatitis, including transient maculopapular rash.

## TRANSMISSION AND PROTECTION

### Transmission

As described previously, HBV is transmitted mainly via percutaneous or permucosal exposure to HBV-containing body fluids. The most critical source of infection is blood (serum) 92. HBV transmission has been found to occur through various forms of human contact, including vertical transmission from mother to newborn, sexual contact, close household contact, needle sharing, and occupational (healthcare) exposure (horizontal transmission) 10. HBV transmission can result from the accidental inoculation of small amounts of blood or other body fluids during medical procedures 6. Nowadays, blood transfusion and organ transplantation are extremely rare routes for HBV transmission. This section will primarily focus on sexual transmission, which is a common route of HBV infection.

HBV is efficiently transmitted by sexual contact 10. The primary risk factors are unprotected sex with an HBV-infected partner, mainly unvaccinated MSM and heterosexual individuals with multiple sex partners or contact with sex workers 6. MSM have long been known to have high rates of STIs 93. They continue to show higher seroprevalence rates of HBV-related markers than the general population 94. Progression through the infection stages is very rapid, and the immune tolerant stage is sometimes absent 24,95.

Heterosexual transmission is still important, as shown by the 40% transmission rate to nonimmune partners of patients with acute HBV hepatitis or chronic HBV infection 96,97. The seroprevalence rates of HBV-related markers are positively correlated with increasing numbers of current and lifetime heterosexual partners 98,99.

### Protection

#### Behavioral approaches

The 2015 Centers for Disease Control and Prevention (CDC) guidelines describe five major strategies for the prevention and control of STIs (Table 5) 100. For primary prevention, the first approach is to change the sexual behavior that can increase the risk of STIs. Information on sexual behavior that can increase the risk of STIs should be provided tactfully. In addition, adolescents and young adults should be made aware that some of the information on protection against STIs may be inaccurate 100. Correcting misinformation on protection against STIs may also reduce the incidence of high-risk sexual behavior^101^. One of the most reliable methods for preventing an STI is refraining from sexual contact, which includes oral, vaginal, and anal sex 100.

**Table 5:** Major strategies for prevention and control of STIs 100. Abbreviation: STIs, sexually transmitted infections.

**Major strategies for prevention and control of STIs.**
Accurate risk assessment and education and counseling of individuals at risk on ways to avoid STIs through changes in sexual behavior and use of recommended devices of prevention;
Pre-exposure vaccination of individuals at risk for vaccine-preventable STIs;
Identification of asymptomatically infected individuals and individuals with symptoms associated with STIs;
Effective diagnosis, treatment, counseling, and follow up of infected individuals;
Evaluation, treatment, and counseling of sex partners of individuals who are infected with an STI.

Over the past 10 years, condom use by unprotected heterosexuals has increased in the United States, suggesting that information on the prevention of STIs is being widely disseminated and understood 102. Additionally, possible sexual partners should be tested for STIs before sexual contact is initiated 100. If one partner has an STI or his/her infection status is unknown, a new condom should be used for each sexual contact.

In summary, safe sex practices, including minimizing the number of sex partners and using barrier protection, can reduce the risk of HBV infection.

#### Hepatitis B immune globulin (HBIG) and hepatitis B (HB) vaccine

Both HBIG and HB vaccines have been approved for preventing HBV infection 103,104.

HBIG is prepared from human plasma containing a high concentration of anti-HBs and provides short-term (3 to 6 months) protection from HBV infection. It is typically used as post-exposure prophylaxis along with HB vaccination for individuals who have never been vaccinated or who have not responded to HB vaccination. The recommended dose of HBIG is 0.06 mL/kg 100.

HB vaccines contain HBsAg that is produced by a recombinant yeast strain 105. Epidemiologic studies have not found any evidence of an underlying association between HB vaccination and sudden infant death syndrome or other causes of death during the first year of life 106,107. Thus, HB vaccination can be considered safe.

HB vaccination is the most effective method of preventing HBV infection 108. The introduction of universal HB vaccination for newborns has been reported to be a very reasonable and cost-effective strategy 109,110. The World Health Organization (WHO) has now included HB vaccination in the Expanded Program on Immunization 22. WHO recommends that all infants receive HB vaccine as soon as possible after birth, preferably within 24 hours. In 2013, 183 WHO member states immunized infants against HBV as a part of their routine vaccination schedule, and 81% of children received HB vaccines 47.

HB vaccine is available for younger children, adolescents, and healthy adults 2. In adolescents and healthy adults (aged younger than 40 years), approximately 30% to 55% of recipients achieve protective antibody responses (i.e., anti-HBs ≥10 mIU/mL) after the first vaccination, 75% after the second, and over 90% after the third. Therefore, HB vaccination can be thought to induce protective antibody response (anti-HBs ≥ 10 mIU/mL) in the majority of recipients. Regardless of the specific patient considerations needed when an HB vaccination schedule is selected, a complete vaccine series should be administered^100^. Recommendations on the HB vaccine dosage and schedule vary, depending on the product used and the recipient’s age 2. Details on HB vaccination are described in guidelines 6,104.

HB vaccine-induced immune memory has been established to last for more than 20 years 111,112,113. According to the 2015 CDC guidelines, periodic monitoring of anti-HBs levels after routine HB vaccination is not needed, and booster doses of HB vaccine are not currently recommended 2. However, the American Red Cross report suggests that HB-vaccine-induced immune memory might be limited; although HB vaccination can prevent clinical liver injury (hepatitis), 100% of subclinical infections cannot be prevented 114. Indeed, although HB vaccine is sufficiently effective at preventing the development of clinical disease (hepatitis), it cannot prevent 100% of HBV infections, resulting in detectable anti-HBc 114. Additionally, there is a report of acute hepatitis B infection in a patient who received five HB vaccinations 115. An MSM patient, who received several HB vaccinations and showed an anti-HBs serological response of >10 mIU/mL (accepted threshold for protection), was reported to have developed a chronic HBV genotype F infection 116. These cases suggest that monitoring anti-HBs levels after routine vaccination might be necessary for certain patients. When the anti-HBs level is too low to provide protection from HBV infection (anti-HBs <10 mIU/mL), a booster vaccination should be administered. Although HB vaccines are highly immunogenic, postvaccination serologic testing might be indicated for infants whose mothers were infected at delivery, individuals with occupational exposure to blood, sexually active individuals such as MSM, or immunosuppressed individuals 10.

#### Pre-exposure vaccination

In 1992, WHO recommended that all countries should introduce universal HB vaccination into their routine immunization programs 117. HB vaccination is recommended for all unvaccinated children and adolescents, all unvaccinated adults with risk of HBV infection (especially MSM, adults with multiple sex partners, and drug users), and all adults desiring protection from HBV infection 104. HB vaccine should be routinely offered to all unvaccinated persons who attend STI clinics or seek evaluation or treatment for STIs in other settings, especially correctional facilities, facilities providing treatment and prevention services for substance use disorder, and settings serving MSM (e.g., HIV care and prevention settings) 2.

#### Postexposure prophylaxis

Both passive-active postexposure prophylaxis (simultaneous administration of HBIG and HB vaccine at separate sites) and active postexposure prophylaxis (administration of HB vaccination alone) have been demonstrated to be highly effective for preventing HBV infection 103. Unvaccinated individuals or those known not to have received a complete HB vaccine series should receive both HBIG and HB vaccine as soon as possible (preferably ≤24 hours) after exposure to blood or body fluids containing HBsAg. HB vaccine should be administered at the same time as HBIG, but at a separate injection site; and the HB vaccine series should be completed, using the age-appropriate vaccine dose and schedule 2. Individuals with certification that they received a complete HB vaccine series and who have never undergone post-vaccination serologic testing should receive a single vaccine booster dose. These individuals should be treated according to guidelines for the management of individuals with occupational exposure to blood or body fluids that contain HBV 118.

## TREATMENT AND CURABILITY

### Treatment

The primary treatment goals for patients with HBV infection are preventing the progression to severe liver disease. The prevention of cirrhosis, hepatic failure, and HCC are most important. The risk factors for progression of chronic HBV include male gender, older age, family history of HCC, elevated alpha-fetoprotein (AFP) level, and coinfection with other viruses (HCV, HDV, or HIV) 119.

For the best outcome, a synergistic approach that decreases the viral load and uses immunotherapeutic interventions to boost the immune response is needed 120. The prevention of HCC often includes antiviral treatment using pegylated interferon (PEG-IFN) or nucleos(t)ide analogues (NAs), which are described later 121.

#### Overall management for types of HBV infection

Patients with acute hepatitis B are treated by supportive care, with no specific treatment. Patient care is focused on maintaining comfort and adequate nutritional balance, including replacement of fluids lost from vomiting and diarrhea. Patients with chronic HBV infection should be referred for evaluation to a provider experienced in the management of chronic HBV infection 122. A variety of treatment algorithms have been proposed, including ones from the American Association for the Study of Liver Diseases (AASLD) 28, the European Association for the Study of the Liver (EASL) 23, the Asian Pacific Association for the Study of the Liver (APASL) 123, the Canadian Association for the Study of the Liver (CASL) 124, and the National Institute for Health and Clinical Excellence (NICE) 125.

In general, for patients with chronic HBV infection who are positive for serum HBeAg, treatment is advised when the serum level of HBV DNA is at or greater than 20,000 IU/mL (10^5^ copies/mL) (or >2,000 IU/mL [EASL recommendation]) and the serum ALT level is elevated (>20 U/L for females and 30 U/L for males) for 3-6 months. For patients with chronic HBV infection who are negative for HBeAg, treatment is advised when the serum level of HBV DNA is at or greater than 2,000 IU/mL (10^4 ^copies/mL) and the serum ALT is elevated (>20 U/L for females and 30 U/L for males) for 3-6 months 126.

Treatment of HIV infection with nucleos(t)ide analogs active against HBV greatly improves the outcomes of hepatic disease, including cirrhosis and HCC, in HIV-HBV coinfected patients, especially when tenofovir is part of the antiviral regimen 127. HBV/HDV coinfection should be treated with PEG-IFN therapy 128.

The National Institutes of Health (NIH) also advises that immediate therapy is not usually indicated for the following patients: 1) patients who are in the immune-tolerant stage, with chronic hepatitis B and high serum levels of HBV DNA but normal serum ALT levels or little activity on liver biopsy; 2) patients who are in an inactive chronically infected/low replicative stage and have low serum levels of or undetectable HBV DNA and normal serum ALT levels; and 3) patients who are not immunosuppressed and have latent HBV infection, defined as detection of HBV DNA in the absence of HBsAg 126.

#### Pharmacologic management

The therapeutic agents cleared by FDA for the treatment of chronic hepatitis B can achieve sustained suppression of HBV replication and remission of liver disease 122. Currently, PEG-IFN-α 2a, entecavir, and tenofovir are available for the treatment of HBV infection. These are the main treatments that have been approved worldwide. Lamivudine, telbivudine, and adefovir are now "nonpreferred" agents, and considered to be of historical interest 129.

#### Pegylated interferon alpha 2a (PEG-IFN-α 2a)

IFNs are naturally produced cytokines. They induce direct antiviral activity by stimulating the host’s antiviral immune response and mediating conflicting effects on viral replication. PEG-IFN-α 2a has a longer half-life and enhanced efficacy relative to standard IFN-α. Pegylation lowers the rate of absorption following subcutaneous injection, reduces renal clearance, and decreases the immunogenicity of IFN 130.

The advantages of IFN therapy are the absence of viral resistance, the finite course of treatment (normally 48 weeks), an increased chance of sustained virological response (SVR), and HBeAg and HBsAg clearance; compared with patients treated by NAs 131. A 48-week regimen of PEG-IFN-α 2a has been found to induce HBeAg seroconversion in 27% of patients and disappearance of serum HBV DNA in 25% of patients 130. Long-term studies have demonstrated that IFN treatment is associated with a significant reduction in the risk of cirrhosis and HCC, even in patients who fail to clear HBeAg 24. However, IFN has a poor side-effect profile (including persistent flu-like symptoms and psychiatric complications) compared with NAs, requires subcutaneous injection, and is not recommended for patients with decompensated cirrhosis 25. HBV genotypes A and D are important and independent predictors of IFN responsiveness in patients with chronic hepatitis B 132. IFN treatment is more effective for patients who are most likely to benefit, especially younger patients, who have more potential years in which to develop complications from their chronic hepatitis B infection and thus have more to gain from achieving an SVR 25,95.

#### Nucleos(t)ide analogues (NAs)

The NIH recommends nucleos(t)ide therapy for the treatment of HBV-infected patients with acute liver failure as well as for cirrhotic patients who are positive for serum HBV DNA; and for patients with clinical complications, cirrhosis, advanced fibrosis with serum positive for HBV DNA, or reactivation of chronic HBV during or after chemotherapy or immunosuppression 126.

Entecavir, a guanosine nucleoside analogue, is a first-line agent for the treatment of HBV infection 51. It is a powerful inhibitor of HBV polymerase. It competes with the natural substrate, deoxyguanosine triphosphate (dGTP), to inhibit HBV polymerase (reverse transcriptase) activity. The advantages of therapy with this agent include potent antiviral activity and a low rate of resistance to the drug 51, although entecavir is used less frequently than other agents for the treatment of lamivudine-resistant HBV.

Results of a retrospective study indicated that assessment of serum HBV DNA levels 12 months after initiation of entecavir therapy may be useful for evaluating entecavir therapy for NA-naïve, HBV-infected patients 7. Investigators found 3 independent predictors for viral suppression lasting 3 years after the start of entecavir therapy: the lowest level of HBV DNA that can be detected, undetectable serum HBV DNA at month 12, and seronegative for HBeAg at the start of therapy. Serum HBV DNA undetectable at month 12 also increased the probability of HBeAg seroconversion and lowered the risk of drug resistance 133.

In another study, after 240 weeks of continuous entecavir therapy for HBeAg-positive patients, 94% of patients had less than 300 copies/mL of serum HBV DNA, and 80% had normal ALT levels. An additional 23% of patients achieved HBeAg seroconversion, and HBsAg disappeared in 1.4% of the patients. Only 1 patient developed resistance to treatment with entecavir within 5 years of treatment 134.

Another study found that long-term treatment with entecavir (about 6 years of cumulative therapy [range, 267-297 weeks]) for NA-naïve patients with chronic HBV infection and advanced fibrosis or cirrhosis, resulted in durable virologic suppression, continued histologic improvement, and reversal of fibrosis or cirrhosis 135.

Tenofovir is the newest antiviral agent. It is a nucleotide-analogue (adenosine monophosphate) inhibitor of viral reverse transcriptase. It may be used as first-line therapy for treatment-naïve patients 51. Patients who received tenofovir continuously for 240 weeks had sustained suppression of serum HBV DNA levels (less than 400 copies/mL). The rate of viral suppression in patients negative for HBeAg was 83%, and in patients positive for HBeAg was 65%. Of the patients positive for HBeAg who received tenofovir through 240 weeks, the rate of disappearance of HBsAg was 9%, and the HBsAg seroconversion rate was 7%. The rate of disappearance of HBeAg was 46%, and the HBeAg seroconversion rate was 40%. There was no evidence of resistance to tenofovir over the treatment period 125. Of note, follow up of the same cohort revealed that after 4.5 years of tenofovir, 87% had histological improvement, 51% had regression of fibrosis, and 74% of the patients with cirrhosis at baseline were no longer cirrhotic 136.

#### Monitoring considerations

The tests that should be used and the frequency of testing will depend on the patient’s serological profile (HBeAg-positive or -negative) and HBV DNA viral levels 126. Patients with chronic active hepatitis should undergo blood testing (aminotransferase levels, HBV status, viral load, and AFP levels), as well as treatment.

For individuals with inactive chronic HBV infection, the current guidelines recommend monitoring for serum HBV DNA and ALT levels at least annually 23,28,137. Patients with cirrhosis must be monitored for HCC by determination of AFP levels every 6 to 12 months and undergoing surveillance by abdominal ultrasonography 28,137. Note, however, that determination of AFP levels was excluded from the AASLD guidelines 28.

### Curability

Despite notable progress regarding many aspects of HBV infection, particularly with respect to prevention and treatment, chronic HBV infection remains strictly noncurable, because residual HBV cccDNA can always be detected in the liver, even after clearance of HBsAg and development of anti-HBs in the serum 138. Moreover, HBV DNA sequences can integrate into the hepatocyte genome, as demonstrated in individuals seronegative for HBsAg 139. Therefore, the term "cure" cannot be used to indicate that HBV is completely eradicated.

There is ongoing discussion on the meaning of "cured’ of chronic HBV infection". The primary treatment goal for HBV infection is to improve patient quality of life and reduce the risk of death from liver disease 140. Large cohort studies of patients with chronic hepatitis B have found a 15% to 40% cumulative risk of developing cirrhosis. Two to five percent of patients with established cirrhosis will develop HCC 141. Three possible types of cure are identified, which are described below 140.

"Absolute cure" means that the patient is free of HBV. That is, there are no HB virions and cccDNA anywhere in the body, including hepatocytes. The patient recovers to the degree of health and medical condition prior to HBV infection, and the probability of developing cirrhosis or HCC depends on age and gender. Although this type of cure is uncommon, it is the most desirable 140.

"Functional cure" means that HBV progression can be controlled. The patient recovers to his or her state of health equal to that of a person who has recovered spontaneously from HBV infection. Both have a similar likelihood of developing cirrhosis or HCC. Current therapy can only achieve "functional cure" through suppression of HBV replication 140.

"Apparent virologic cure" is defined as a sustained off-drug suppression of virologic markers and the normalization of liver function. This last definition includes an SVR, which is the ongoing suppression of viral load following the cessation of therapy, and adds the disappearance of all circulating viral markers (seroclearance), and the possible suppression of cccDNA. A complicating factor with HBV infection is that patients who have achieved a serologic resolution of infection (loss of HBsAg, undetectable serum HBV DNA, appearance of anti-HBs) can develop reactivation of their disease because of immunosuppression or the use of anti-inflammatory medications 142,143. In addition, it is important to note that in occult HBV infection, despite the complete loss of HBsAg and undetectable or very low levels of HBV DNA in serum, there may still be an increased risk for progression to cirrhosis and the development of HCC 144.

An "apparent virologic cure" or "functional cure" as a desirable endpoint for therapy is supported by a recent study looking at the risk of HCC in patients with or without spontaneous seroclearance of HBV seromarkers 145. However, none of these endpoints turned out to be a reliable indicator of favorable long-term outcome of chronic HBV infection. Thus, for the time being, HBsAg loss is viewed as the best possible predictor of a favorable long-term outcome of HBV infection and is used as an endpoint 146,147.

## CONCLUSIONS

HBV infection is one of the common STIs having a major worldwide impact on a patient’s clinical health status and on public health, and is also associated with liver-related morbidity and mortality. New HBV infections in industrialized countries are becoming increasingly concentrated among individuals at risk for STIs, infants, and injection drug users. HB vaccines have been an effective prevention strategy for individuals at risk through sexual exposure, especially MSM and heterosexuals with multiple sex partners. The proper education of persons at risk for STIs may also help with their acceptance of HB vaccination.

Regarding the persistence of HB vaccine-induced immunity, the effectiveness of routine HB vaccination might not last long enough to prevent 100% of HBV infections. In our opinion, postvaccination serologic testing, especially for anti-HBs, should be introduced for groups at high risk for HBV infection, after careful consideration. Potential causes of vaccine failure, such as infection with HBV variants, require further study. The need for booster doses to preserve vaccine-induced immunity should be evaluated regularly, especially for infants whose mothers were infected, individuals with occupational risk, sexually active individuals such as MSM, or individuals under immunosuppression.
